# Diagnostic value of serum NSE, and S-100β and inflammatory markers levels in epilepsy

**DOI:** 10.5937/jomb0-55343

**Published:** 2026-02-27

**Authors:** Zhaoxia Li, Li Su, Yi Lu, Yunqing Hu, Qian Yang

**Affiliations:** 1 The First Affiliated Hospital of Bengbu Medical University, Department of Neurology, Bengbu, China; 2 Lixin County People's Hospital, Department of Laboratory Medicine, Bozhou, China; 3 Haikou Fourth People's Hospital, Department of Neurology, Haikou, China

**Keywords:** IL-2, IL-8, TNF-α, serum NSE, s-100β, cytokines, secondary epilepsy, sodium valproate, IL-2, IL-8, TNF-α, serumski NSE, s-100β, citokini, sekundarna epilepsija, natrijum-valproat

## Abstract

**Background:**

To sodium valproate lore the effects of sodium valproate on serum inflammatory factors and s-100<span style="color: rgb(32, 33, 34); font-family: sans-serif; font-size: 16px; background-color: rgb(248, 249, 250);">β</span> levels in patients with emergency secondary epilepsy(SE).

**Methods:**

This was a retrospective cohort of 120 patients with SE who received Sodium valproate compared with a group who received carbamazepine for different therapeutic drugs. The general data, interleukin (IL)-2, IL-8, tumour necrosis factor (TNF)-<span style="color: rgb(32, 33, 34); font-family: sans-serif; font-size: 16px; background-color: rgb(248, 249, 250);">α</span>, neuron-specific enolase (NSE), serum acid calcium-binding protein s-100<span style="color: rgb(32, 33, 34); font-family: sans-serif; font-size: 16px; background-color: rgb(248, 249, 250);">β</span>, total effective rate (TER), seizure onset condition, and adverse reactions (Ars) of the two groups were compared at baseline and at 3 months later.

**Results:**

There were no significant differences in age, gender, BMI, course of the disease, stroke type, or seizure type between the study groups (P&gt; 0.05). The results showed that IL-2 of the Sodium valproate group (53.17± 4.95 <span style="color: rgb(32, 33, 34); font-family: sans-serif; font-size: 16px; background-color: rgb(248, 249, 250);">μ</span>g/L) was lower versus Carbamazepine group (62.38± 4.83 <span style="color: rgb(32, 33, 34); font-family: sans-serif; font-size: 16px; background-color: rgb(248, 249, 250);">μ</span>g/L) (P&lt; 0.05). The IL-8 of the Sodium valproate group postoperatively (26.48± 2.73 <span style="color: rgb(32, 33, 34); font-family: sans-serif; font-size: 16px; background-color: rgb(248, 249, 250);">μ</span>g/L) was lower versus the Carbamazepine group (33.54± 3.39 <span style="color: rgb(32, 33, 34); font-family: sans-serif; font-size: 16px; background-color: rgb(248, 249, 250);">μ</span>g/L) (P&lt; 0.05). Postoperatively, the TNF-<span style="color: rgb(32, 33, 34); font-family: sans-serif; font-size: 16px; background-color: rgb(248, 249, 250);">α</span> in Sodium valproate group (32.18± 4.26 <span style="color: rgb(32, 33, 34); font-family: sans-serif; font-size: 16px; background-color: rgb(248, 249, 250);">μ</span>g/L) was lower versus the Carbamazepine group (41.03± 4.92 <span style="color: rgb(32, 33, 34); font-family: sans-serif; font-size: 16px; background-color: rgb(248, 249, 250);">μ</span>g/L) (P&lt; 0.05). The s-100<span style="color: rgb(32, 33, 34); font-family: sans-serif; font-size: 16px; background-color: rgb(248, 249, 250);">β</span> (0.29 ± 0.15 ) <span style="color: rgb(32, 33, 34); font-family: sans-serif; font-size: 16px; background-color: rgb(248, 249, 250);">μ</span>g/L in the Sodium valproate group was lower versus the Carbamazepine group (0.54± 0.14) <span style="color: rgb(32, 33, 34); font-family: sans-serif; font-size: 16px; background-color: rgb(248, 249, 250);">μ</span>g/L (P &lt; 0.05). The TER of the Sodium valproate group (93.33% ) was higher versus the Carbamazepine group of 75% (P &lt; 0 .0 5 ). The number of epileptic seizures in the Sodium valproate group was 0.81± 0.08 times per year versus 1.23± 0.12 times per year in the Carbamazepine group. The duration of epilepsy in the Sodium valproate group (2.53± 0.22 min/time) was shorter than that in the Carbamazepine group (3.08 ± 0.24 min/time).

**Conclusions:**

Sodium valproate can drastically relieve the epileptic symptoms of patients with SE, and there was no great difference in ARs. Hence, it is safe and worthy of popularization and application.

## Abstract (sr)

**Uvod:**

Cilj je bio da se ispita efekat natrijum-valproata na serumske inflamatorne faktore i nivoe s-100<span style="color: rgb(32, 33, 34); font-family: sans-serif; font-size: 16px; background-color: rgb(248, 249, 250);">β</span> kod pacijenata sa urgentnom sekundarnom epilepsijom (SE).

**Metode:**

Retrospektivna kohortna studija je obuhvatila 120 pacijenata sa SE koji su lečeni natrijum-valproatom i grupu koja je primala karbamazepin kao terapiju. Upoređivani su opšti podaci, interleukini (IL-2, IL-8), faktor nekroze tumora (TNF)-<span style="color: rgb(32, 33, 34); font-family: sans-serif; font-size: 16px; background-color: rgb(248, 249, 250);">α</span>, neuronski specifična enolaza (NSE), serumski kalcijum-vezujuci protein s-100<span style="color: rgb(32, 33, 34); font-family: sans-serif; font-size: 16px; background-color: rgb(248, 249, 250);">β</span>, ukupna efikasnost terapije (TER), učestalost epileptičnih napada i neželjene reakcije (AR) između dve grupe na početku i nakon tri meseca.

**Rezultati:**

Nisu zabeležene značajne razlike u godinama, polu, BMI, trajanju bolesti, tipu moždanog udara ili tipu napada između grupa (P&gt; 0,05). Rezultati su pokazali da je IL-2 u grupi sa natrijum-valproatom (53,17 ± 4,95 <span style="color: rgb(32, 33, 34); font-family: sans-serif; font-size: 16px; background-color: rgb(248, 249, 250);">μ</span>g/L) bio niži u poređenju sa grupom sa karbamazepinom (62,38± 4,83 <span style="color: rgb(32, 33, 34); font-family: sans-serif; font-size: 16px; background-color: rgb(248, 249, 250);">μ</span>g/L) (P&lt; 0,05). IL-8 u grupi sa natrijum-valproatom postoperativno (26,48± 2,73 <span style="color: rgb(32, 33, 34); font-family: sans-serif; font-size: 16px; background-color: rgb(248, 249, 250);">μ</span>g/L) je bio niži u poređenju sa grupom sa karbamazepinom (33,54± 3,39 <span style="color: rgb(32, 33, 34); font-family: sans-serif; font-size: 16px; background-color: rgb(248, 249, 250);">μ</span>g/L) (P&lt; 0,05). Postoperativno, TNF-<span style="color: rgb(32, 33, 34); font-family: sans-serif; font-size: 16px; background-color: rgb(248, 249, 250);">α</span> u grupi sa natrijum-valproatom (32,18± 4,26 <span style="color: rgb(32, 33, 34); font-family: sans-serif; font-size: 16px; background-color: rgb(248, 249, 250);">μ</span>g/L) je bio niži u poređenju sa grupom sa karbamazepinom (41,03± 4,92 <span style="color: rgb(32, 33, 34); font-family: sans-serif; font-size: 16px; background-color: rgb(248, 249, 250);">μ</span>g/L) (P&lt; 0,05). Nivo s-100<span style="color: rgb(32, 33, 34); font-family: sans-serif; font-size: 16px; background-color: rgb(248, 249, 250);">β</span> (0,29 ± 0,15 <span style="color: rgb(32, 33, 34); font-family: sans-serif; font-size: 16px; background-color: rgb(248, 249, 250);">μ</span>g/L) u grupi sa natrijum-valproatom je bio niži u poređenju sa grupom sa karbamazepinom (0,54 ± 0,14 <span style="color: rgb(32, 33, 34); font-family: sans-serif; font-size: 16px; background-color: rgb(248, 249, 250);">μ</span>g/L) (P&lt; 0,05). Ter u grupi sa natrijum-valproatom (93,33% ) je bio viši u poređenju sa grupom sa karbamazepinom (75%) (P&lt; 0,05). Broj epileptičnih napada u grupi sa natrijum-valproatom je bio 0,81± 0,08 puta godišnje u poređenju sa 1,23± 0,12 puta godišnje u grupi sa karbamazepinom. Trajanje napada u grupi sa natrijum-valproatom (2,53± 0,22 min/napad) bilo je kraće nego u grupi sa karbamazepinom (3,08± 0,24 min/napad).

**Zaključak:**

Natrijum-valproat značajno ublažava simptome epilepsije kod pacijenata sa SE, a razlike u AR nisu bile velike. Stoga je bezbedan i koristan za širu primenu.

## Introduction

Epilepsy is a common chronic condition within the field of functional neurosurgery. It is primarily caused by the abnormal release of epileptic waves from brain neuron cells, leading to transient and recurrent abnormalities in brain function [1]. When epilepsy occurs without an identifiable cause, it is referred to as idiopathic epilepsy [2]. In contrast, epilepsy arising from specific underlying conditions is termed secondary epilepsy (SE) [3]. The causes of SE often include intracranial tumours, craniocerebral trauma, cerebral haemorrhage, subarachnoid haemorrhage, intracranial infections, and various toxic encephalopathies [4]. Compared to primary epilepsy, SE is predominantly associated with lesions in brain tissue. Advances in clinical examination techniques have revealed that SE accounts for more than 90% of all epilepsy cases. Treatment strategies for SE vary and should be tailored to the specific circumstances of each patient [5][6].

Epileptic disorders result from neuronal damage in the brain. While medications are generally used to control seizures, surgery is an option when pharmacological treatments prove ineffective. SE can arise from numerous factors, including cerebral infarction, cerebral haemorrhage, encephalitis, and carbon monoxide toxic encephalopathy, often resulting in recurrent seizures [7]. Sodium valproate and carbamazepine are widely used antiepileptic drugs for managing SE following a stroke [8]. Sodium valproate is a broad-spectrum antiepileptic medication effective for various seizure types, including simple partial seizures, complex partial seizures, myoclonic seizures, and generalized seizures [9][10]. It can be administered as monotherapy or in combination with other drugs [11].

Sodium valproate, a histone deacetylase inhibitor, has been shown to regulate macrophage polarization by suppressing inflammatory responses and promoting the production of anti-inflammatory cytokines. These effects suggest its therapeutic potential in treating diseases associated with macrophage dysfunction [12]. Although sodium valproate is a well-established treatment for SE [13], its underlying mechanisms are not yet fully understood. Research indicates that sodium valproate reduces inflammation in bovine mammary epithelial cells by inhibiting the NOD1-NF-κB pathway and histone modifications, thereby decreasing the production of pro-inflammatory cytokines [14]. Additionally, valproic acid has been found to reduce inflammation and promote neuroprotection by modulating microglial polarization and inhibiting the NF-κB pathway via STAT1 acetylation, which depends on histone deacetylase 3 (HDAC3) activity [15].

A meta-analysis demonstrated that individuals with epilepsy exhibit significantly elevated serum levels of S100B, a calcium-binding protein, compared to healthy controls, indicating its potential as a diagnostic and prognostic biomarker for epilepsy [16]. Furthermore, elevated serum S-100β levels have been shown to reliably indicate delayed neurological sequelae caused by decreased oxygenation in the brain [17].

Hence, this work was developed to explore the therapeutic effect and related mechanism of sodium valproate by measuring the changes in serum inflammatory factors and acid calcium-binding protein S-100β levels in patients with SE treated with sodium valproate. This study aims to provide a reference for the clinical treatment of SE by highlighting sodium valproate's anti-inflammatory and neuroprotective effects, emphasizing its potential to improve therapeutic outcomes for SE patients. The novelty of this study lies in its focus on the comparative evaluation of sodium valproate and carbamazepine, particularly in the context of emergency secondary epilepsy, which has been less extensively explored in existing literature.

## Materials and methods

### Study design and population

In this retrospective study, 120 patients with secondary epilepsy (SE) admitted to Haikou Fourth People's Hospital were included. Patients treated with sodium valproate were compared with those receiving carbamazepine, with 60 patients in each group. Patients in the sodium valproate group received sodium valproate as their treatment, while those in the carbamazepine group were administered carbamazepine. Inclusion criteria were: i) patients with a history of stroke and a diagnosis of SE, ii) patients with no history of antiepileptic drug use in the previous month, and iii) patients with complete clinical data. Exclusion criteria included: i) patients with primary epilepsy, ii) patients with primary secretory diseases, iii) patients with mental illness, and iv) patients who were non-cooperative.

### Interventions

A total of 120 patients with SE were divided into a sodium valproate group and a carbamazepine group, each consisting of 60 patients. The carbamazepine group received carbamazepine tablets twice daily, escalating to two tablets twice daily after two weeks. The sodium valproate group was treated with one tablet of sodium valproate three times daily, increasing to two tablets three times daily after two weeks. Peripheral venous blood samples were collected within 12 hours of a seizure for serum preparation. Serum interleukin (IL)-2, IL-8, and tumour necrosis factor (TNF)-α levels were measured using a double-antibody sandwich enzyme-linked immunosorbent assay (ELISA). Venous blood samples were collected before treatment and three months after reexamination, with serum obtained through low-temperature centrifugation. Serum neuron-specific enolase (NSE) and S-100β levels were also measured by ELISA. General patient data, changes in serum inflammatory factors, S-100β levels, seizure frequency, and adverse reactions (ARs) were recorded pre- and post-treatment. These findings were analyzed to evaluate the clinical efficacy of sodium valproate in treating SE and to provide guidance for clinical management.

### Evaluation of the curative effect

The curative effect was categorized into four levels based on symptom control and improvement [18]: complete control, significantly effective, effective, and invalid, as detailed in Table 1.

**Table 1:** Classification of epilepsy treatment effect.

Type	Criteria for evaluation
Complete controlled	During the follow-up period, the patient had no further epileptic symptoms.
Significantly effective	During the follow-up period, the number of seizures decreased by more than 3/4 versus that before treatment.
Effective	During the follow-up treatment, the number of seizures was reduced by 1/2 to 3 /4 versus that before treatment.
Invalid	The improvement in the number of seizures was less than 1/2 before treatment.

### Observed indicators

The following indicators were observed and analyzed:

- General patient data, including sex, age, body mass index (BMI), disease duration, stroke type, and seizure type.
- Serum inflammatory factors, including IL-2, IL-8, TNF-α, serum NSE, and S-100β levels.
- Efficacy evaluation grades, including complete control, significantly effective, effective, invalid, and the total effective rate (TER) [18].
- Seizure characteristics, such as seizure frequency and duration.
- Adverse reactions include rash, somnolence, headache, insomnia, nausea and vomiting, paresthesia, and abnormal liver function.

### Statistical analysis

Statistical analysis was conducted using SPSS 19.0 software. Measurement data were expressed as mean±standard deviation (x̄±s). The independent samples t-test was used for comparisons between groups, and the paired t-test was applied for pre- and post-treatment comparisons. Count data were expressed as percentages (%) and analyzed using the χ^2^ test. A P-value <0.05 was considered statistically significant.

## Results

### Characteristics of patients included

The sodium valproate group included 35 males and 25 females, with an average age of 62.35±6.83 years (range: 55-70 years). The carbamazepine group comprised 33 males and 27 females, with an average age of 63.01 ±7.24 years (range: 56-71 years). There were no significant differences in general characteristics such as sex, age, BMI, disease duration, or seizure type between the two groups (P>0.05), indicating that the groups were comparable in baseline demographics and clinical features (Table 2).

**Table 2:** The contrast of general data.

Type	Sodium valproate group<br>(60 cases)	Control group<br>(60 cases)	t/χ^2^	P
Sex			0.338	0.564
Male	35	33		
Female	25	27		
Age (years old)	62.35±6.83	63.01±7.24	0.751	0.398
BMI (kg/m^2^)	24.62±3.23	24.74±3.09	-0.571	0.615
Course of disease (years)	4.26±1.45	4.33±1.38	0.625	0.426
Stroke types			0.369	0.724
Cerebral infarction	39	37		
Cerebral hemorrhage	21	23		
Type of epilepsy			0.459	0.547
General tonic attack	29	26		
Simple partial attack	15	17		
Complex partial attack	10	11		
Mixed attack	6	6		

### Serum inflammatory cytokines

In both groups, levels of inflammatory markers, including IL-2, IL-8, and TNF-α, significantly decreased after treatment (P<0.05). Pre-treatment levels of IL-2 were similar in the sodium valproate group (90.05±11.52 μg/L) and the carbamazepine group (90.13±10.96 μg/L, P>0.05). However, post-treatment IL-2 levels were significantly lower in the sodium valproate group (53.17±4.95 μg/L) compared to the carbamazepine group (62.38±4.83 μg/L, P<0.05). Similar trends were observed for IL-8 and TNF-α. Post-treatment IL-8 levels in the sodium valproate group (26.48±2.73 μg/L) were significantly lower than in the carbamazepine group (33.54±3.39 μg/L, P<0.05). TNF-α levels also decreased more markedly in the sodium valproate group (32.18±4.26 μg/L) compared to the carbamazepine group (41.03±4.92 μg/L, P<0.05, Table 3).

**Table 3:** Comparison of inflimatory factors.

Marker	Group	Baseline	Follow-up	P-value<br>(vs. Baseline)	P-value<br>(vs. Ctrl)
** IL-2 **	** Sodium valproate **	90.05±11.52	53.17±4.95	<0.05	<0.05
	** carbamazepine **	90.13±10.96	62.38±4.83	<0.05	-
** IL-8 **	** Sodium valproate **	47.51±5.57	26.48±2.73	<0.05	<0.05
	** carbamazepine **	46.82±5.49	33.54±3.39	<0.05	-
** TNF-α **	** Sodium valproate **	69.95±9.93	32.18±4.26	<0.05	<0.05
	** carbamazepine **	69.44±9.51	41.03±4.92	<0.05	-
** NSE **	** Sodium valproate **	23.06±3.95	14.46±1.47	<0.05	<0.05
	** carbamazepine **	22.19±3.88	18.22±2.11	<0.05	-
** S-100β **	** Sodium valproate **	1.31±0.35	0.73±0.23	<0.05	<0.05
	** carbamazepine **	1.34±0.32	0.92±0.19	<0.05	-

### Serum NSE and S-100β levels

Serum levels of NSE and S-100β significantly decreased in both groups following treatment (P<0.05). Pre-treatment NSE levels were comparable between the sodium valproate group (23.06±3.95 μg/L) and the carbamazepine group (22.19±3.88 μg/L, P>0.05). After one month of treatment, NSE levels were significantly lower in the sodium valproate group (14.53±1.47 μg/L) compared to the carbamazepine group (18.22±2.11 μg/L, P<0.05). This trend persisted at three months, with NSE levels in the sodium valproate group (11.46±0.65 μg/L) remaining lower than in the carbamazepine group (11.95±0.87 μg/L, P<0.05). Similarly, S-100β levels decreased significantly post-treatment in both groups. At one month, the sodium valproate group showed lower S-100β levels (0.73±0.23 μg/L) than the carbamazepine group (0.92±0.19 μg/L, P<0.05). This difference widened further at three months, with levels of 0.29±0.15 μg/L in the sodium valproate group compared to 0.54±0.14 μg/L in the carbamazepine group (P<0.05, Table 3).

### Treatment efficacy

The sodium valproate group demonstrated a higher total effective rate (TER) of 93.33% compared to 75% in the carbamazepine group (P<0.05). In the sodium valproate group, 17 patients achieved complete control, 25 were significantly effective, and 9 were effective. In contrast, the carbamazepine group had 14 cases of complete control, 20 significantly effective cases, and 7 effective cases. These findings indicate that sodium valproate was more effective overall in managing secondary epilepsy (Table 4).

**Table 4:** Comparison of study outcomes among groups.

Group	TER (%)	Seizures/Year<br>(Pre)	Seizures/Year<br>(Post)	Seizure<br>Duration (Pre)	Seizure<br>Duration (Post)	ARs (n)	ARs (%)
** Sodium valproate **	93 .33	2.69±0.24	0.81±0.08	4.53±0.61	2.53±0.22	16	26.67
** Carbamazepine **	75	2.68±0.21	1.23±0.12	4.49±0.58	3.08±0.24	14	23.33

### Seizure frequency and duration

Both groups experienced significant reductions in seizure frequency and duration after treatment (P<0.05). In the sodium valproate group, the number of seizures decreased from 2.69±0.24 times per year pre-treatment to 0.81±0.08 times per year post-treatment. In the carbamazepine group, seizure frequency reduced from 2.68±0.21 times per year to 1.23±0.12 times per year. Post-treatment seizure frequency was significantly lower in the sodium valproate group (P<0.05). Similarly, the average duration of seizures decreased from 4.53±0.61 minutes to 2.53±0.22 minutes in the sodium valproate group and from 4.49±0.58 minutes to 3.08±0.24 minutes in the carbamazepine group. The sodium valproate group exhibited shorter seizure durations compared to the carbamazepine group post-treatment (P<0.05, Table 4).

### Adverse reactions

The incidence of adverse reactions (ARs) was slightly higher in the sodium valproate group (26.67%) compared to the carbamazepine group (23.33%). Common ARs in the sodium valproate group included rash (3 cases), somnolence (2 cases), insomnia (1 case), nausea and vomiting (4 cases), paraesthesia (3 cases), and abnormal liver function (3 cases). The carbamazepine group reported rash (3 cases), somnolence (3 cases), headache (1 case), nausea and vomiting (4 cases), and paraesthesia (5 cases). Both treatments were generally well tolerated, with no severe adverse events recorded (Table 5).

**Table 5:** The contrast of the occurrence of ARs.

Group	Rash	Somnolence	Headache	Insomnia	Nausea and	Paresthesia	Abnormal liver
Control group	3	3	1		4	5	
Sodium valproate	3	2		1	4	3	3

## Discussion

Our study evaluated the effects of sodium valproate on serum inflammatory factors and S-100β levels in patients with emergency secondary epilepsy (SE), comparing its outcomes with carbamazepine. The results demonstrate that sodium valproate significantly reduced IL-2, IL-8, TNF-α, and S-100β levels, indicating robust anti-inflammatory and neuroprotective effects. Additionally, sodium valproate achieved a higher total effective rate and significantly reduced seizure frequency and duration compared to carbamazepine. These findings suggest sodium valproate's efficacy in managing SE, an area where targeted treatment options are limited.

In contrast, the Cochrane Review [18][19] reported no significant difference between carbamazepine and valproate monotherapy for epilepsy in terms of time to treatment withdrawal, 12-month remission, and time to first seizure. However, it highlighted that carbamazepine might be more effective for partial epilepsies, whereas valproate may be more beneficial for generalized epilepsies. Our focus on emergency secondary epilepsy - a distinct clinical entity - underscores the need for specialized treatment strategies that might differ from those used in broader epilepsy contexts.

Interestingly, Gomez et al. [20] observed that valproic acid (VPA) failed to reduce IL-1β and TNF-α expression in the rat hippocampus, whereas carbamazepine (CBZ) demonstrated significant anti-inflammatory effects. However, our findings show that sodium valproate effectively reduced IL-2, IL-8, and TNF-α levels in human patients with SE. The discrepancies may arise from species-specific differences in pharmacokinetics, pharmacodynamics, and cytokine expression profiles, emphasizing the challenges of translating preclinical findings to clinical practice.

Our study differs from Shehata et al. [21], which assessed the neuropsychological effects of carbamazepine versus valproate in adult males with epilepsy. While direct comparisons are challenging due to differing methodologies and outcomes, both studies highlight the importance of understanding antiepileptic drugs (AEDs) in terms of their broader impact on patients' health.

The findings of Zhong et al. [18] align closely with our study, reporting reduced S-100β levels following treatment with valproic acid [16]. Conversely, Bayat et al. [22] found that CBZ therapy reduced the risk of metabolic syndrome, while valproic acid therapy increased it. Although our study did not evaluate metabolic outcomes, the association between metabolic syndrome and inflammation highlights the potential interplay between systemic health and AED therapy. Differences in populations, study designs, and methods of measuring inflammation may account for these variations.

Tao et al. [13] further support our findings, showing that sodium valproate combined with lamotrigine improved quality of life and reduced serum inflammatory factors in post-stroke SE patients. These findings reinforce the anti-inflammatory and neuroprotective effects of valproate sodium. Its mechanism of action involves inhibiting histone deacetylase (HDAC), increasing histone acetylation, and suppressing pro-inflammatory gene expression. Valproate sodium has also been shown to inhibit nuclear factor kappa B (NF-κB), reducing the production of pro-inflammatory cytokines such as TNF-α and IL-1β. Additionally, it alleviates neuropathic pain and spinal neuroinflammation by downregulating cytokines like TNF -a, IL-1 b, and IL-6, inhibiting microglial activation, and promoting neuroprotection by reducing spinal cell apoptosis [23].

The relatively small sample size and single-centre design may limit the generalizability of the findings. Additionally, the retrospective nature of the study introduces potential biases, and the short follow-up period does not account for long-term efficacy and safety. While inflammatory markers and S-100β levels were measured, their direct correlation with clinical outcomes was not fully explored. Furthermore, potential confounding factors such as comorbidities and genetic predispositions were not controlled.

## Conclusion

Sodium valproate in the treatment of SE can drastically relieve the epilepsy symptoms of patients and drastically reduce the levels of s-100β and inflammatory factors, and there was no great difference in ARs. Therefore, sodium valproate is safe and worth popularizing and applying. However, due to the limited research objects and scope of this study, it is necessary to further sodium valproate and the research scope in future research.

## Dodatak

### Acknowledgment

None.

### Financial support

None.

### Authors contribution

Zhaoxia Li conceptualized and designed the study, supervised the research process, and contributed to manuscript preparation. Li Su participated in data collection, analysis, and interpretation of results. Yi Lu provided critical insights into the methodology and assisted in refining the study design. Qian Yang contributed to the acquisition of clinical data and ensured the accuracy of statistical analyses. Yunqing Hu supported the study by contributing to the literature review, assisting in drafting the manuscript and reviewing it critically for intellectual content. All authors read and approved the final manuscript, ensuring its scientific accuracy and integrity.

### Conflict of interest statement

All the authors declare that they have no conflict of interest in this work.

## References

[1] Klein P, Kaminski R M, Koepp M, Löscher W (2024). New epilepsy therapies in development. Nature Reviews Drug Discovery.

[2] Sen A, Jette N, Husain M, Sander J W (2020). Epilepsy in older people. The Lancet.

[3] Royer J, Bernhardt B C, Larivière S, Gleichgerrcht E, Vorderwülbecke B J, Vulliémoz S, et al (2022). Epilepsy and brain network hubs. Epilepsia.

[4] Milligan T A (2021). Epilepsy: A Clinical Overview. The American Journal of Medicine.

[5] Asadi-Pooya A A, Brigo F, Lattanzi S, Blumcke I (2023). Adult epilepsy. The Lancet.

[6] Balestrini S, Arzimanoglou A, Blümcke I, Scheffer I E, Wiebe S, Zelano J, et al (2021). The aetiologies of epilepsy. Epileptic Disorders.

[7] Falco-Walter J (2020). Epilepsy-Definition, Classification, Pathophysiology, and Epidemiology. Seminars in Neurology.

[8] Zelano J, Holtkamp M, Agarwal N, Lattanzi S, Trinka E, Brigo F (2020). How to diagnose and treat post-stroke seizures and epilepsy. Epileptic Disorders.

[9] Billakota S, Devinsky O, Kim K - W (2020). Why we urgently need improved epilepsy therapies for adult patients. Neuropharmacology.

[10] Ding D, Zhou D, Sander J W, Wang W, Li S, Hong Z (2021). Epilepsy in China: major progress in the past two decades. The Lancet Neurology.

[11] Dean J C, Penry J K (2020). The Medical Treatment of Epilepsy.

[12] Wu C, Li A, Leng Y, Li Y, Kang J (2012). Histone Deacetylase Inhibition by Sodium Valproate Regulates Polarization of Macrophage Subsets. DNA and Cell Biology.

[13] Tao S, Sun J, Hao F, Tang W, Li X, Guo D, et al (2020). Effects of Sodium Valproate Combined with Lamotrigine on Quality of Life and Serum Inflammatory Factors in Patients with Poststroke Secondary Epilepsy. Journal of Stroke and Cerebrovascular Diseases.

[14] Gao Q, Wang Y, Ma N, Dai H, Roy A C, Chang G, et al (2020). Sodium valproate attenuates the iE-DAP induced inflammatory response by inhibiting the NOD1-NF-κB pathway and histone modifications in bovine mammary epithelial cells. International Immunopharmacology.

[15] Chen S, Ye J, Chen X, Shi J, Wu W, Lin W, et al (2018). Valproic acid attenuates traumatic spinal cord injury-induced inflammation via STAT1 and NF-κB pathway dependent of HDAC3. J Neuroinflammation.

[16] Liang K - G, Mu R - Z, Liu Y, Jiang D, Jia T - T, Huang Y - J (2019). Increased Serum S100B Levels in Patients With Epilepsy: A Systematic Review and Meta-Analysis Study. Front Neurosci.

[17] Hafez A S A F, El-Sarnagawy G N (2019). S-100β in predicting the need of hyperbaric oxygen in CO-induced delayed neurological sequels. Human & Experimental Toxicology.

[18] Zhong G, Liang L, Chen X, Zhong G, Huang C, Lin P, et al (2024). Therapeutic Impact of Serum Inflammatory Cytokines and S-100 Levels in Patients With Acute Secondary Epilepsy Treated With Sodium Valproate. American Journal of Therapeutics.

[19] Marson A G, Williamson P R, Hutton J L, Clough H E, Chadwick D W (2000). Carbamazepine versus valproate monotherapy for epilepsy. The Cochrane Database of Systematic Reviews.

[20] Gómez C D, Buijs R M, Sitges M (2014). The anti-seizure drugs vinpocetine and carbamazepine, but not valproic acid, reduce inflammatory IL-1β and TNF-α expression in rat hippocampus. Journal of Neurochemistry.

[21] Shehata G A, Bateh AE - aM, Hamed S A, Rageh T A, Elsorogy Y B (2009). Neuropsychological effects of antiepileptic drugs (carbamazepine versus valproate) in adult males with epilepsy. Neuropsychiatr Dis Treat.

[22] Bayat M, Jalali N, Poursadeghfard M, Nazeri M, Dabbaghmanesh M H, Ashjazadeh N (2021). Frequency of metabolic syndrome and insulin resistance in epileptic patients treated with sodium valproate or carbamazepine monotherapy: A Case-Control Study.

[23] Stakišaitis D, Kapočius L, Tatarūnas V, Gečys D, Mickienė A, Tamošuitis T, et al (2024). Effects of Combined Treatment with Sodium Dichloroacetate and Sodium Valproate on the Genes in Inflammation- and Immune-Related Pathways in T Lymphocytes from Patients with SARS-CoV-2 Infection with Pneumonia: Sex-Related Differences. Pharmaceutics.

